# A Combined-Radiomics Approach of CT Images to Predict Response to Anti-PD-1 Immunotherapy in NSCLC: A Retrospective Multicenter Study

**DOI:** 10.3389/fonc.2021.688679

**Published:** 2022-01-10

**Authors:** Minghao Wu, Yanyan Zhang, Jianing Zhang, Yuwei Zhang, Yina Wang, Feng Chen, Yahong Luo, Shuai He, Yulin Liu, Qian Yang, Yanying Li, Hong Wei, Hong Zhang, Nian Lu, Sicong Wang, Yan Guo, Zhaoxiang Ye, Ying Liu

**Affiliations:** ^1^ Department of Radiology, Beijing Tiantan Hospital, Capital Medical University, Beijing, China; ^2^ Department of Radiology, Tianjin Medical University Cancer Institute and Hospital, National Clinical Research Center for Cancer, Key Laboratory of Cancer Prevention and Therapy, Tianjin’s Clinical Research Center for Cancer, Tianjin, China; ^3^ Department of Medical Oncology, 1st Affiliated Hospital, Zhejiang University School of Medicine, Hangzhou, China; ^4^ Department of Radiology, 1st Affiliated Hospital, Zhejiang University School of Medicine, Hangzhou, China; ^5^ Department of Medical Imaging, Cancer Hospital of China Medical University, Liaoning Cancer Hospital and Institute, Shenyang, China; ^6^ Department of Radiology, Hubei Cancer Hospital, Tongji Medical College, Huazhong University of Science and Technology, Wuhan, China; ^7^ Department of Thoracic Oncology, Cancer Center, West China Hospital, Sichuan University, Chengdu, China; ^8^ Department of Radiology, West China Hospital, Sichuan University, Chengdu, China; ^9^ Department of Radiology, Tianjin Chest Hospital, Tianjin, China; ^10^ Department of Radiology, Sun Yat-sen University Cancer Center, State Key Laboratory of Oncology in Southern China, Guangzhou, China; ^11^ Prognostic Diagnosis, GE Healthcare China, Beijing, China

**Keywords:** immunotherapy, non-small-cell lung cancer, radiomics, computed tomography, response prediction

## Abstract

**Objective:**

Based on non-contrast-enhanced (NCE)/contrast-enhanced (CE) computed tomography (CT) images, we try to identify a combined-radiomics model and evaluate its predictive capacity regarding response to anti-PD1 immunotherapy of patients with non-small-cell lung cancer (NSCLC).

**Methods:**

131 patients with NSCLC undergoing anti-PD1 immunotherapy were retrospectively enrolled from 7 institutions. Using largest lesion (LL) and target lesions (TL) approaches, we performed a radiomics analysis based on pretreatment NCE-CT (NCE-radiomics) and CE-CT images (CE-radiomics), respectively. Meanwhile, a combined-radiomics model based on NCE-CT and CE-CT images was constructed. Finally, we developed their corresponding nomograms incorporating clinical factors. ROC was used to evaluate models’ predictive performance in the training and testing set, and a DeLong test was employed to compare the differences between different models.

**Results:**

For TL approach, both NCE-radiomics and CE-radiomics performed poorly in predicting response to immunotherapy. For LL approach, NCE-radiomics nomograms and CE-radiomics nomograms incorporating with clinical factor of distant metastasis all showed satisfactory results, reflected by the AUCs in the training (AUC=0.84, 95% CI: 0.75-0.92; AUC=0.77, 95% CI: 0.67-0.87) and test sets (AUC=0.78, 95% CI: 0.64-0.92, AUC=0.73, 95% CI: 0.57-0.88), respectively. Compared with the NCE-radiomics nomograms, the combined-radiomics nomogram showed incremental predictive capacity in the training set (AUC=0.85, 95% CI: 0.77-0.92) and test set (AUC=0.81, 95% CI: 0.67-0.94), respectively, but no statistical difference (*P*=0.86, *P*=0.79).

**Conclusion:**

Compared with radiomics based on single NCE or CE-CT images, the combined-radiomics model has potential advantages to identify patients with NSCLC most likely to benefit from immunotherapy, and may effectively improve more precise and individualized decision support.

## Introduction

Immunotherapy has revolutionized the therapeutic strategies for non-small cell lung cancer (NSCLC) (1–4). More recently, immune checkpoint inhibitors (ICIs) antibodies targeting the PD-(L)1 axis have revolutionized cancer treatment and improved long-term survival among some patients with locally advanced and metastatic NSCLC (5, 6). Unfortunately, only a small proportion (20-50%) of patients with advanced solid tumors respond to immunotherapy (7–9). Moreover, due to multiple mechanisms of immunotherapy (10), atypical patterns produced by immunotherapy (e.g., durable and/or delayed responses, pseudoprogression, and hyperprogression) cannot be adequately captured by traditional response criteria (11, 12). As immunotherapy is expensive and could bring serious immune-related adverse events (irAEs, such as pneumonitis), it is necessary to stratify patients according to potential benefit before immunotherapy.

Currently, different biomarkers have been investigated with variable success in the selection of patients eligible for cancer immunotherapy, such as FDA-approved PD-L1 expression, microsatellite instability-high and/or mismatch repair status, and tumor mutation burden (TMB) (13–15). However, due to the intratumoral heterogeneity and evolution over time (16, 17), the effective use of these biomarkers as predictive biomarkers is seriously affected by sampling bias (18) and the absence of standardization between different tests (19). Another issue is that patients with negative PD-L1 status may still benefit from anti-PD(L)1 immunotherapy (4, 20, 21). To better predict response to immunotherapy, there is an urgent need to identify alternative predictive biomarkers.

Radiomics can extract quantitative imaging features in a high-throughput manner and assess tumor microenvironment and heterogeneity (22). In recent years, radiomics-based biomarkers have shown success in predicting response to ICIs (23–27). Nevertheless, previous studies have focused on the role of radiomics based on single CE-CT or NCE-CT images, especially CE-CT images (28, 29). A recent study about the diagnosis of solitary pulmonary nodule indicated that the tumor biological heterogeneity depicted by radiomics features may be confounded by the intravenously injected contrast material (30). However, a study suggested there may be a potential complementary value between CE-CT and NCE-CT radiomics in predicting colorectal cancer survival (31). Currently, it is still unknown whether NCE-CT or CE-CT is more favorable for extracting radiomics features in predicting response to ICIs.

**Figure 1:**
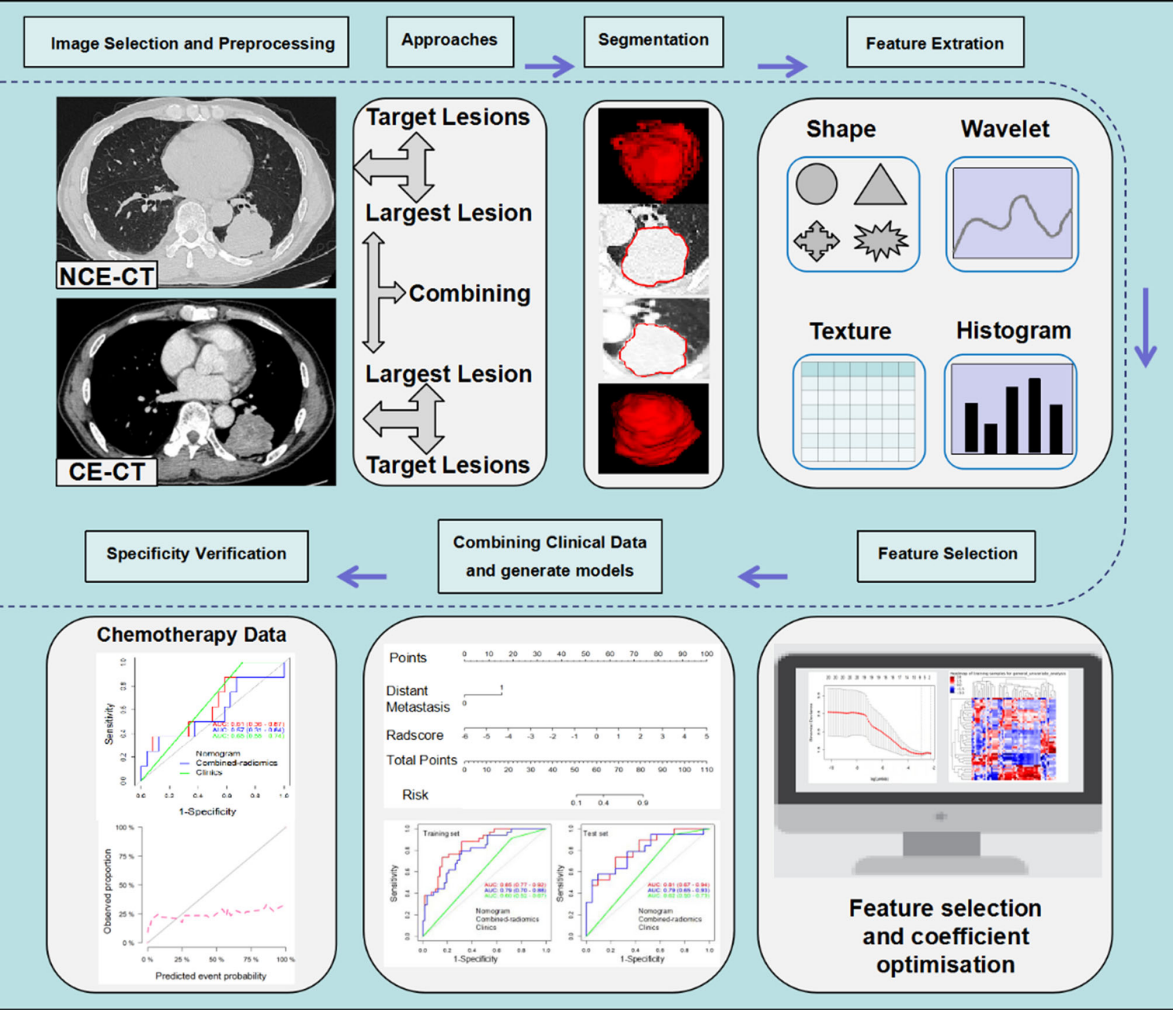
Radiomics workflow. The workflow presents a summary of data collection, study approaches and semi-automatic delineation, modeling schemes of radiomics and specificity verification. NCE-CT, non contrast enhanced CT; CE-CT, contrast enhanced CT.

In the current study, we compared the efficacy of radiomics models based on NCE and CE-CT images from largest target lesion (LL) and target lesions (TL) approaches in predicting response to ICIs in NSCLC, respectively. At the same time, we also developed a combined-radiomics model based on both NCE-CT and CE-CT images from the LL approach to further improve the prediction efficiency. Finally, we aimed to develop and validate combined-radiomics nomograms based on pretreatment NCE-CT and CE-CT images incorporating clinical factors to predict response to NSCLC immunotherapy. The workflow is presented in **Figure 1**.

## Materials and Methods

### Immunotherapy Dataset

**Figure 2:**
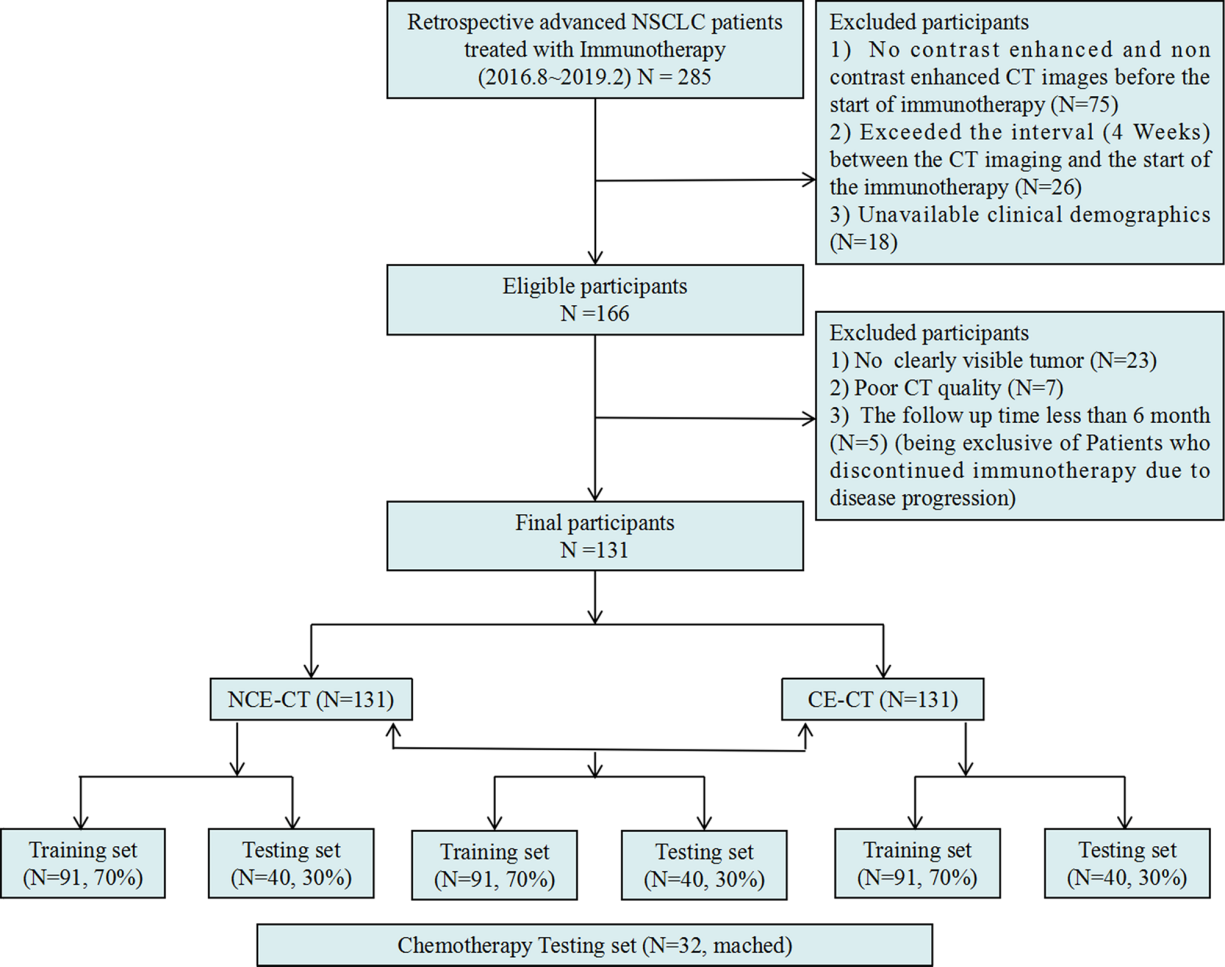
Inclusion and exclusion diagram. Training and testing sets were randomly divided in a proportion of 7:3 respectively as well.

This retrospective multicenter study (NCT04079283) was approved by the institutional ethics committee of each participating hospital, and the requirement for informed consent was waived. This study was performed according with the ethical standards of the Declaration of Helsinki. Initially, a total of 285 patients with advanced NSCLC treated with a PD-1/PD-L1 ICIs therapy (nivolumab/pembrolizumab) from August 1, 2016 to February 28, 2019 in 7 participating institutions were enrolled according to the inclusion criteria (**Appendix S1** and **Figure 2**). According to the exclusion criteria (**Appendix S1** and **Figure 2**), 154 patients were excluded. Finally, the study included the remaining 131 patients. The entire cohort was randomly divided into a training set (n = 92) and an independent testing set (n = 39) at a ratio of 7:3.

### Chemotherapy Dataset

Refer to the previous research (25), to verify the specificity of the radiomic model in predicting response to immunotherapy, we retrospectively collected 32 patients with stage III-IV NSCLC undergoing platinum-based chemotherapy at Tianjin Medical University Cancer Institute and Hospital between 2017 and 2020 as an additional testing set (**Figure 2**), according to the inclusion criteria (**Appendix S2**).

### Images Acquisition and Harmonizing

Pre-treatment NCE-CT and CE-CT images were acquired on a varied set of CT scanners (**Appendix S3**). Because slice thickness images may have adverse effects on radiomics feature extraction (22, 30, 32–34), we preprocessed the images. All CT images were resampled in three directions with a resolution of 1.5 mm to standardize the patient’s voxel size. In addition, Z-score normalization was applied to unify the CT value scale of the scanner.

### Lesion Segmentation and Response Kinetics

Three experienced readers reviewed baseline NCE-CT and CE-CT images and defined the target lesions according to RECIST (35) consensus, and then the largest lesion was chosen from target lesions of each patient by measuring two-dimensional maximum diameter. All target lesions on NCE-CT and CE-CT CT images were manually segmented *via* ITK-SNAP (www.itksnap.org) by two readers. If there is a dispute between two readers, a third reader with 13 years of experience in thoracic radiology will judge and modify it. To narrow the difference in tumor boundaries between the pulmonary window and mediastinal window images, the window width and window level of NCE-CT and CE-CT images were uniformly set at 1200 and -500.

Clinically, immunotherapy response assessment is often performed six months after treatment (23, 36). Therefore, treatment response on images at follow-up six months after therapy according to iRECIST (37) served as the endpoint of our study. Response status was dichotomized as follows: Complete response (iCR), partial response (iPR) or stable disease (iSD) were classified as “response”; Confirmed progressive disease (iCPD) were considered as “non-response”. For patients who were considered to be unconfirmed progression (iUPD) at follow-up 6 months after treatment, their treatment responses needed to be further determined with additional follow-ups to ensure that iUPD would not be used as labels for model training. For the chemotherapy dataset, treatment response was evaluated using RECIST 1.1 (35).

### Feature Engineering and Signature Building

A total of 1316 radiomics features (RFs) were extracted from all target lesions of baseline NCE-CT and CE-CT images of the training set using Artificial Intelligence Kit software version 3.3.0 (GE Healthcare, China) (**Table S1**). Firstly, 107 hand-craft RFs were extracted from CT images, including 24 gray level co-occurrence matrix features, 18 first-order histogram features, 16 gray level size zone matrix features, 16 gray level run length matrix features, 14 shape features, 14 gray level dependence matrix features, 5 neighboring gray tone difference matrix features. After wavelet, LoG and LBP transform based on CT images, 744 wavelet features, 186 laplacian (LoG_sigma_=2.0/3.0) features, and 279 local binary pattern features were obtained respectively. Firstly, Minimum Redundancy Maximum Relevance (mRMR) was used to preprocess these extracted features to remove redundant and irrelevant features. Then, Least Absolute Shrinkage and Selection Operator (LASSO) logistic regression was performed to choose the optimized subset of features. A linear combination of selected features and coefficient vector was used to calculate the radiomics signature (radscore) for each patient. Largest lesion (LL) and target lesions (TL) approaches were used to construct the radiomics modes toward individual-wise analysis. LL approach: radscore of largest target lesion is regarded as individual radiomics signature to predict immunotherapy response; TL approach: average radscore of all target lesions served as a global radiomics signature to predict therapy response. The formula was as follows:


$$\text{Average Radscore}=({\text{Radscore}}_{\text{Lesion }1}+{\text{Radscore}}_{\text{Lesion }2}+{\text{Radscore}}_{\text{Lesion n}})\text{/n }(\text{n}\leq 5)$$

RFs extracted from baseline NCE-CT and CE-CT images were used to construct radiomics model based on NCE-CT image (NCE-radiomics) and radiomics model based on CE-CT image (CE-radiomics), respectively. Finally, the combined-radiomics model based on both NCE-CT and CE-CT images was constructed, and the combined-radscore was calculated by summing the optimal radscores based on NCE-CT and CE-CT images weighted by their coefficients (38).

### Statistical Analysis

The inter-observer reproducibility evaluation of lesion segmentation was quantitatively measured using the Dice Similarity Coefficient. The Chi-square test and Mann-Whitney U test were used to test differences of categorical variables and continuous variables, respectively. To construct the best nomograms incorporating clinical factors and radiomics models, multivariate logistic regression analysis with backward elimination method was employed. The area under the curve of the ROC curve (AUC) and its confidence interval were determined according with the DeLong test. The predictive accuracy of the nomogram was evaluated by calibration curves. Decision curve analysis (DCA) was performed by quantifying the net benefits at different threshold probabilities to evaluate the clinical utility of the radiomics nomogram. *P* values less than 0.05 were regarded as significant. Statistical analyses were performed using R (version 3.5.1) and Python (version 3.5.6).

## Results

### Clinical Characteristics

Among the enrolled 131 patients, a total of 235 target lesions were identified for all patients. The most common lesion site was the lung (n=158, 67%), followed by a small portion of lymph nodes (n=65, 28%) and other organs (n=12, 5%), including liver, adrenal glands, kidneys, and spleen. Among the 131 retrospective patients, 48.85% patients (n=64) showed iPR, 41.22% patients (n=54) showed iPD, and the rest of patients presented iCR (n=3, 2.29%) or iSD (n=10, 7.63%) at the sixth month. The overall disease control rate (DCR) remained at 58.8% (77 of 131). The training set and the test set had identical distributions in clinical characteristics, and the differences were not statistically significant, which proved that they can be used as training set and testing set (**Table 1**). The differences in clinical characteristics between response and non-response were statistically insignificant, except for distant metastasis (*P*=0.001) (**Table 1**). Among the enrolled 32 patients with chemotherapy, the differences of clinical characteristics between response and non-response were not statistically significant (**Table S2**). No significant statistical differences in clinical characteristics were observed between chemotherapy data and training set, except for pathological type (P=0.016) (**Table 2**).

**Table 1:** Baseline clinical characteristics comparison of the 131 cases between training set and testing set, and responders and non-responders.

Variables	Sample	Training set	Testing set	*P Value*	Responders	Non-responders	*P Value*
Age, median		62 (57, 68)	62 (55, 68)	0.554	62 (55, 69)	62 (57, 66)	0.99
Sex, No. (%)				0.915			0.21
Male	112	78 (85.71%)	34 (85.00%)		70 (88.61%)	42 (80.77%)	
Female	19	13 (14.29%)	6 (15.00%)		9 (11.39%)	10 (19.23%)	
Smoking history, No. (%)				0.397			0.49
Non-smokers	36	27 (29.67%)	9 (22.50%)		20 (25.32%)	16 (30.77%)	
Smokers	95	64 (70.33%)	31 (77.50%)		59 (74.68%)	36 (69.23%)	
Pathological type, No. (%)				0.954			0.32
Adenocarcinoma	66	46 (50.55%)	20 (50.00%)		37 (46.84%)	29 (55.77%)	
Others	65	45 (49.45%)	20 (50.00%)		42 (53.16%)	23 (44.23%)	
Distant metastasis, No. (%)				0.655			0.001
Absence	26	19 (20.88%)	7 (17.50%)		23 (29.11%)	3 (5.77%)	
Presence	105	72 (79.12%)	33 (82.50%)		56 (70.89%)	49 (94.23%)	
Treatment strategy, No. (%)				0.903			0.31
Immunotherapy	71	49 (53.85%)	22 (55.00%)		40 (50.63%)	31 (59.62%)	
Combination therapy	60	42 (46.15%)	18 (45.00%)		39 (49.37%)	21 (40.38%)	

**Table 2:** Baseline clinical characteristics comparison of patients between immunotherapy training set and chemotherapy cohorts.

Variables	Sample	Immunotherapy	Chemotherapy	*P* Value
Age, median		62 (56, 68)	60 (55, 65)	0.165
Sex, No. (%)				0.769
Male	102	76 (83.52%)	26 (81.25%)	
Female	21	15 (16.48%)	6 (18.75%)	
Smoking history, No. (%)				0.157
Non-smokers	35	29 (31.87%)	6 (18.75%)	
Smokers	88	62 (68.13%)	26 (81.25%)	
Pathological type, No. (%)				0.016
Adenocarcinoma	70	46 (50.55%)	24 (75.00%)	
Others	53	45 (49.45%)	8 (25.00%)	
Distant metastasis, No. (%)				0.8
Absence	25	18 (19.78%)	7 (21.88%)	
Presence	98	73 (80.22%)	25 (78.12%)	

### Reader Reproducibility

The agreement between the two readers was good. The dice scores of inter-observer lesion segmentation were 0.94 and 0.96 for NCE-CT and CE-CT, respectively.

### Response Status Prediction Nomogram With NCE-RFs or CE-RFs

**Figure 3:**
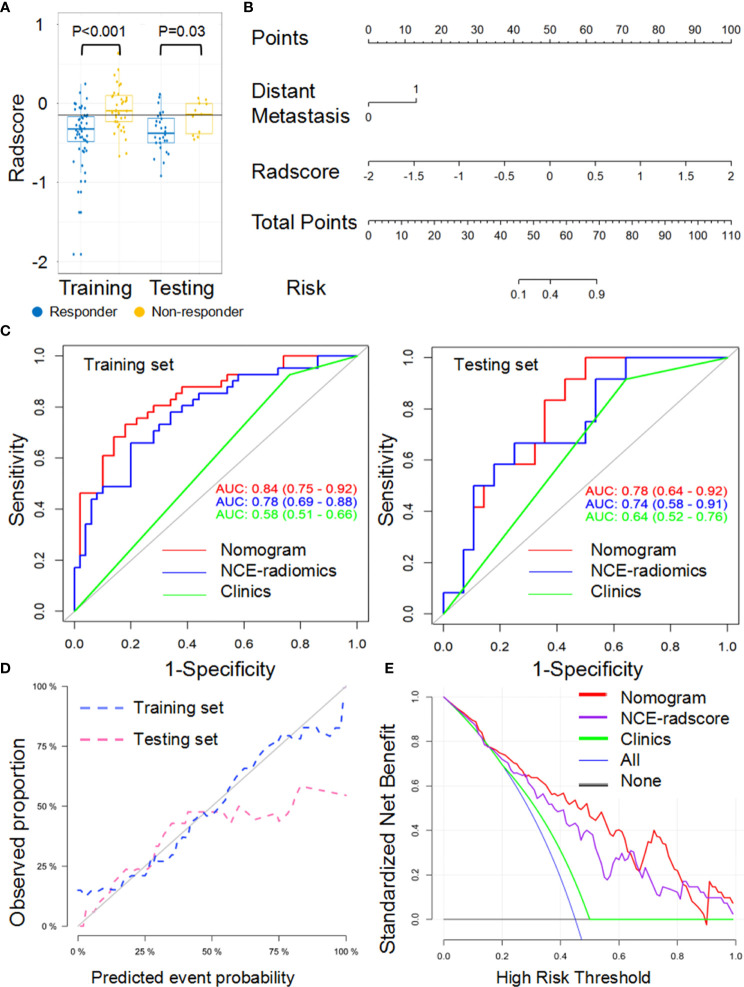
Performance of the NCE-radiomic models from largest lesion approach in training and testing sets. **(A)** Box and whisker plots depict radscore comparison between responders and non-responders. **(B)** NCE-radiomic nomogram developed in training set. **(C)** ROC curves of radiomics signatures in training and testing sets. **(D)** Calibration curve analysis for the nomogram in training set and testing set. **(E)** Decision curve analysis for the nomogram (red), radscore (purple), and clinical model (green). The y-axis indicates the net benefit; x-axis indicates threshold probability. The blue line represents the assumption that all patients were responders. The black line represents the hypothesis that no patients were responders.

**Figure 4:**
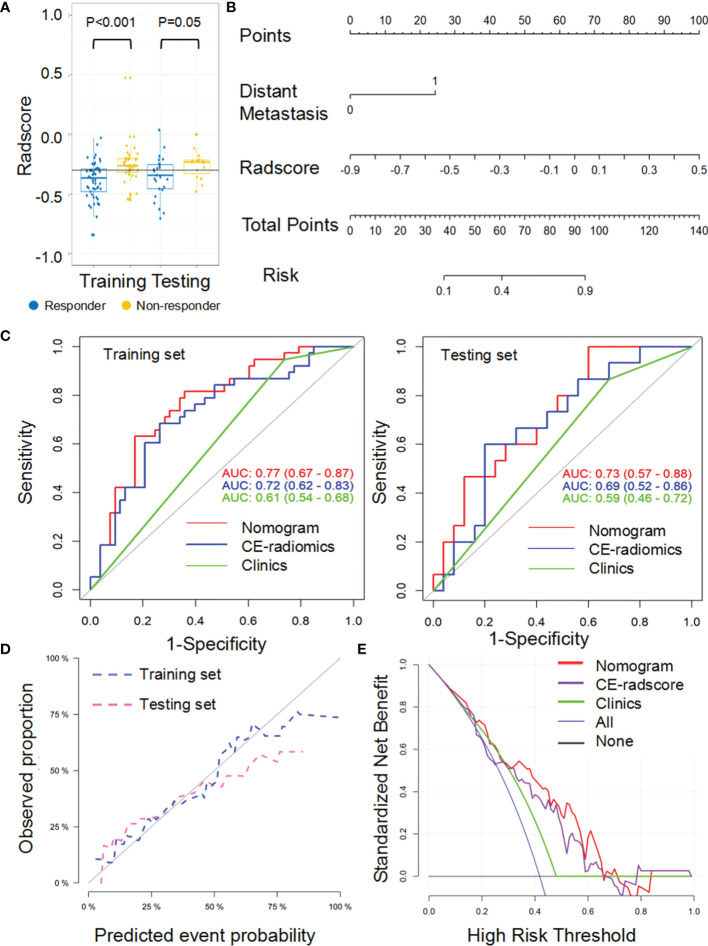
Performance of the CE-radiomic signature from largest lesion approach in training and testing sets. **(A)** Box and whisker plots depict radscore comparison between responders and non-responders. **(B)** Radiomic nomogram developed in training set. **(C)** ROC curves of radiomics signatures in training and testing sets. **(D)** Calibration curve analysis for the nomogram in training set and testing set. **(E)** Decision curve analysis for the nomogram (red), radscore (purple), and clinical model (green). The y-axis indicates the net benefit; x-axis indicates threshold probability. The blue line represents the assumption that all patients were responders. The black line represents the hypothesis that no patients were responders.

For the LL approach, there was a significant difference in radscore from NCE-CT images (NCE-radscore) (Equation 1, **Appendix S5**) between responders and non-responders in the training set (*P*<0.001), which was then confirmed in the testing set (*P*<0.05) (**Figure 3A**). Responders also presented a lower level of radscore from CE-CT images (CE-radscore) (Equation 2, **Appendix S5**) in the training set (*P*<0.001), and the difference was borderline significant in the testing set (*P*=0.05) (**Figure 4A**). The NCE-radiomics signature exhibited significant AUCs value of 0.78 (95% CI, 0.69 to 0.88) and 0.74 (95% CI, 0.58 to 0.91) in the training set and testing set respectively (**Figure 3C**, **Table 3**), as did the CE-radiomics signature (0.72, 95% CI, 0.62 to 0.83; 0.69, 95% CI, 0.52 to 0.86) (**Figure 4C** and **Table 3**). The difference of AUCs for two signatures in training and testing sets all have no significance (*P*=0.44; *P*=0.66). The developed NCE-radiomics nomogram (**Figure 3B**) that combined NCE-radscore with the clinical factor of distant metastasis achieved the highest AUCs of 0.84 (95% CI, 0.75 to 0.92) and 0.78 (95% CI, 0.64 to 0.92) in training and testing sets respectively (**Figure 3C** and **Table 3**). The calibration curves of the NCE-radiomics nomogram (**Figure 3D**) showed good agreements between the nomogram prediction and actual observation in the training set (*P*=0.84) and testing set (*P*=0.16), respectively. The DCA shown in **Figure 3E** indicated that the NCE-radiomics nomogram from the LL approach had the highest overall net benefit across the majority of the range of reasonable threshold probabilities in all the patients compared with NCE-radiomics or clinical signatures alone. The developed CE-radiomics nomogram (**Figure 4B**) also showed a good result in predicting response status with AUCs of 0.77 (95% CI, 0.67 to 0.87) and 0.73 (95% CI, 0.57 to 0.88) in training and testing sets respectively (**Figure 4C** and **Table 3**). The difference of AUC for two nomograms in training and testing sets all have no significance (*P*=0.30; *P*=0.62). The calibration curves of the CE-radiomics nomogram (**Figure 4D**) showed good agreements between the nomogram prediction and actual observation in the training set (*P*=0.54) and testing set (*P*=0.74), respectively. The DCA indicated that the CE-radiomics nomogram (**Figure 4E**) adding more net benefit than the CE-radiomics or clinical signatures alone had a smaller threshold probability range for a patient than the NCE-radiomics nomogram (**Figure 3E**).

**Table 3:** ROC analysis for NCE-radiomics, CE-radiomics and combined-radiomics models from largest lesion approach.

Variables	NCE-radscore	NCE-radiomics nomogram	CE-radscore	CE-radiomics nomogram	Combined-radscore	Combined-radiomics nomogram
Training set						
AUC (95% CI)	0.78 (0.69-0.88)	0.84 (0.75-0.92)	0.72 (0.62-0.83)	0.77 (0.67-0.87)	0.79 (0.70-0.88)	0.85 (0.77-0.92)
Specificity	0.80	0.79	0.74	0.76	0.68	0.84
Sensitivity	0.66	0.77	0.68	0.73	0.79	0.74
Accuracy (95% CI)	0.74 (0.63-0.82)	0.78 (0.68-0.86)	0.71 (0.61-0.80)	0.75 (0.65-0.83)	0.73 (0.62-0.81)	0.80 (0.71-0.88)
Testing set						
AUC (95% CI)	0.74 (0.58-0.91)	0.78 (0.64-0.92)	0.69 (0.52-0.86)	0.73 (0.57-0.88)	0.79 (0.65-0.93)	0.81 (0.67-0.94)
Specificity	0.79	1.00	0.56	1.00	0.67	0.76
Sensitivity	0.58	0.46	0.73	0.50	0.63	0.74
Accuracy (95% CI)	0.73 (0.56-0.85)	0.65 (0.48-0.79)	0.62 (0.46-0.77)	0.63 (0.46-0.77)	0.65 (0.48-0.79)	0.75 (0.59-0.87)

For the TL approach, two NCE-RFs and seven CE-RFs were selected through the LASSO logistic regression analysis (**Figure S1Aa** and **Figure S1Ba**). The radscore of non-responders was slightly higher than responders in the training set (*P*=0.008) from NCE-radscore (Equation 3, **Appendix E4**), but did not reach a significant difference in the testing set (*P*=0.23) (**Figure S1Ab**). The NCE-radiomics signature carried out no superior prediction value in training and testing sets (AUC=0.66, 95% CI, 0.55 to 0.78; AUC=0.63, 95% CI, 0.41 to 0.85) (**Figure S1Ac** and **Table 4**). The CE-radscore (Equation 4, **Appendix S5**) was significantly higher in nonresponders than in responders in the training set (*P*<0.001), and the difference was slightly significant in the testing set (*P*=0.04) (**Figure S1Bb**). The CE-radiomics signature exhibited AUCs value of 0.75 (95% CI, 0.65 to 0.85) and 0.68 (95% CI: 0.49-0.88) in training and testing sets respectively (**Figure S1Bc** and **Table 4**).

**Table 4:** ROC analysis for the NCE-radiomics and CE-radiomics models from target lesions approach.

Variables	NCE-radscore		CE-radscore	
	Training set	Testing set	Training set	Testing set
AUC (95% CI)	0.66 (0.55-0.78)	0.63 (0.41-0.85)	0.75 (0.65-0.85)	0.68 (0.49-0.88)
*P*	0.008	0.23	<0.001	0.041
Specificity	0.70	0.65	0.67	0.52
Sensitivity	0.61	0.64	0.72	0.61
Accuracy (95% CI)	0.66 (0.55-0.76)	0.65 (0.48-0.79)	0.69 (0.59-0.78)	0.55 (0.38-0.71)

### Combined-Radiomics Nomogram Building and Evaluation With Both NCE-CT and CE-CT Radscore

**Figure 5:**
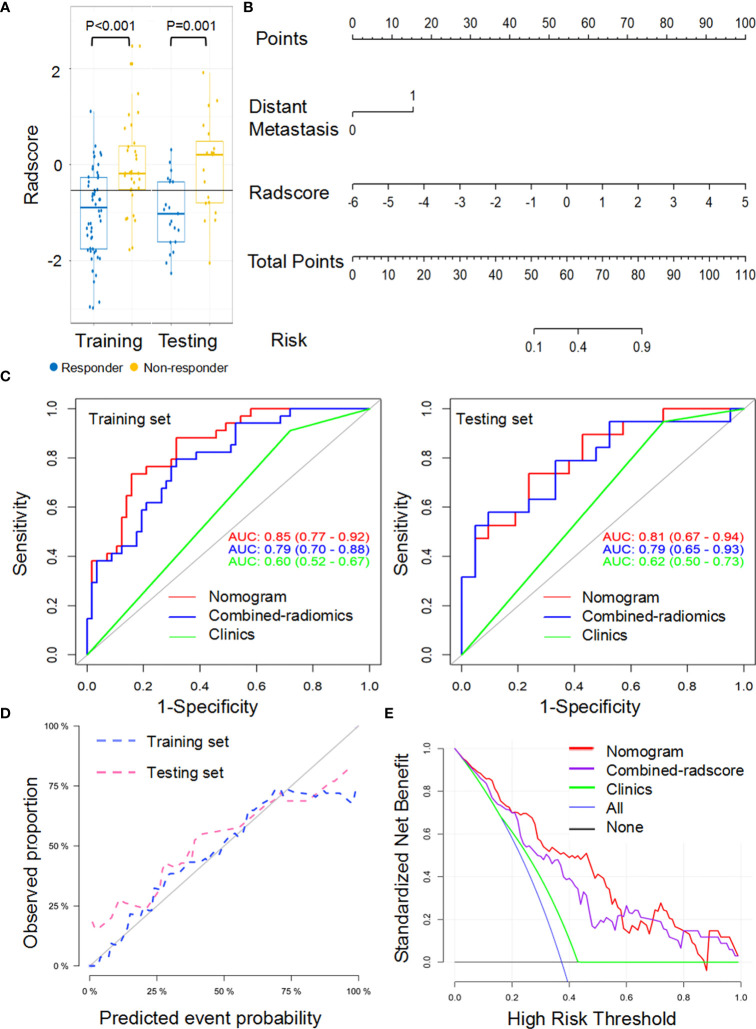
Performance of the combined-radiomic signature from largest lesion approach in training and testing sets. **(A)** Box and whisker plots depict radscore comparison between responders and non-responders. **(B)** Radiomic nomogram developed in training set. **(C)** ROC curves of radiomics signatures in training and testing sets. **(D)** Calibration curve analysis for the nomogram in training set and testing set. **(E)** Decision curve analysis for the nomogram (red), radscore (purple), and clinical model (green). The y-axis indicates the net benefit; x-axis indicates threshold probability. The blue line represents the assumption that all patients were responders. The black line represents the hypothesis that no patients were responders.

Because radiomics models from the TL approach did not exhibit a higher predictive value than that from the LL approach, combined-radscore was calculated by summing the NCE-radscore and CE-radscore weighted by their coefficients from the LL approach (Equation 5, **Appendix E4**). There was a significant statistical difference in combined-radscore between responders and non-responders in the training set (*P*<0.001) and testing set (*P*=0.003), respectively (**Figure 5A**). The combined-radiomics model yielded significantly strong prediction results with an AUC of 0.79 (95% CI, 0.77 to 0.92) in the training set and 0.79 (95% CI, 0.67 to 0.94) in the testing set (**Figure 5C** and **Table 3**). This combined-radiomics model did perform better prediction performance in the testing set than the NCE-radiomics model and CE-radiomics model, but the improvement did not reach significance in the Delong Test (*P*=0.67, *P*=0.37, respectively). Interestingly, the specificity and sensitivity of the model to predict immunotherapy responses were optimized by combining NCE-CT and CE-CT images (**Table 3**).

A combined-radiomics nomogram (**Figure 5B**) which incorporated the combined-radiomics model based on NCE-CT and CE-CT images with the clinical factor of distant metastasis was chosen as the best response status classifier. The combined-radiomics nomogram showed significantly strong prediction results with an AUC of 0.83 (95% CI, 0.75-0.91) in the training set and an AUC of 0.81 (95% CI, 0.69-0.93) in the testing set (**Figure 5C**). The difference of AUC between combined-radiomics nomogram and NCE-radiomics nomogram in training and testing sets all have no significance (*P*=0.86, *P*=0.79). The prediction accuracy of the nomogram was 0.75 in the testing set (**Table 3**). The calibration curves of the combined-radiomics nomogram (**Figure 5D**) showed good agreements between the nomogram prediction and actual observation in the training set (*P*=0.81) and testing set (*P*=0.58), respectively. The DCA indicated that the combined-radiomics nomogram from the LL approach had the highest overall net benefit across the majority of the range of reasonable threshold probabilities in all the patients compared with combined-radiomics or clinical signatures alone (**Figure 5E**).

### Proposed Combined-Radiomics Features as a Set of ICI-Specified Biomarkers

**Figure 6:**
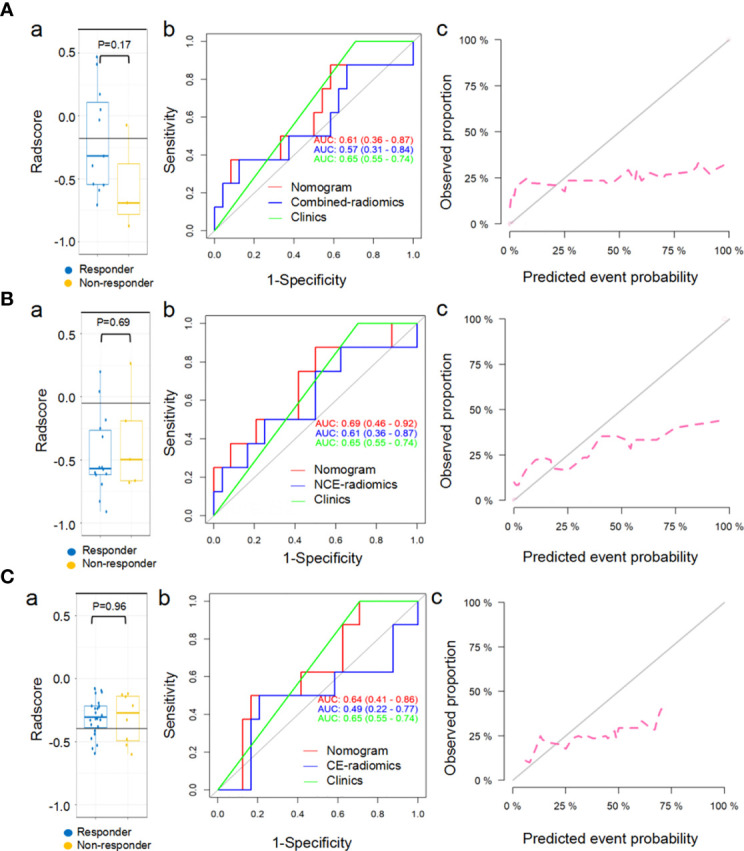
Predictive performance of combined-radiomic **(A)**, NCE-radiomic **(B)** and CE-radiomic **(C)** models in chemotherapy cohorts. **(Aa, Ba, Ca)** Box and whisker plots depict radscore comparison between responders and non-responders. **(Ab, Bb, Cb)** ROC curves of radiomics signatures in chemotherapy cohorts. **(Ac, Bc, Cc)** Calibration curve analysis for the nomograms in chemotherapy cohorts.

The proposed combined-radscore (**Figure 6Aa**) did not show any significant predictive value in predicting response status of chemotherapy at the sixth month (AUC=0.57, *P*=0.17) (**Figure 6Ab** and **Table S3**), nor did NCE-radiomics and CE-radiomics signature (AUC=0.61, *P*=0.98; AUC=0.49, *P*=0.69) (**Figures 6B, C** and **Table S3**). The developed combined-radiomics nomogram, NCE-radiomics nomogram, and CE-radiomics nomogram still achieved low AUCs (**Figures 6Ab, Bb, Cb** and **Table S3**). The calibration curves of the three nomograms (**Figures 6Ac, Bc, Cc**) all showed very poor agreements between the nomogram prediction and actual observation in chemotherapy cohorts (*P*<0.001).

## Discussion

In recent years, radiomics studies of immunotherapy mainly extracted features from CE-CT images (25, 26, 28, 29), but few studies from NCE-CT images. However, it has been suggested that NCE-CT images is more conducive to the feature extraction of radiomics (30). We first compared the NCE-radiomics model with the CE-radiomics model in predicting response to immunotherapy. NCE-radiomics model showed better performance in predicting response to immunotherapy, which was inconsistent with prior studies (25). NCE-radiomics nomogram showed better performance compared with CE-radiomics nomogram. The underlying reason may be that contrast material injected in intravenous had adverse effects on the extraction of radiomics features depicting the biological heterogeneity within the tumor (39–41). In addition, the scanning scheme was preset, and different patients have different hemodynamic characteristics, so the RFs based on CT value were affected to some extent. Although the original images were preprocessed, the problem could not be fundamentally solved. Our results were similar to those observed in a previous study (30) which showed that NCE-radiomics signature was more informative on the differential diagnosis of the solitary pulmonary nodule (SPN) than CE-radiomics signature.

Typically, radiomics studies of lung cancer focused on extracting features from a primary lung tumor, largest lung lesion, or one of the target lesions (23, 26, 27, 29). By contrast, target lesions were all included in our analysis. We assumed that the TL approach which was more consistent with clinical RECIST1.1 could reflect total tumor burden to some extent. Under the circumstance of a mix-response pattern (present both responding and progressive lesions), the TL approach could avoid potential selection bias compared with the LL approach. However, in our study, radiomics models for the TL approach did exhibit lower predictive value. A previous study suggested that the predictive performance of the radiomics model from all lesions on metastases from different organs is different (25). Such a discrepancy can be explained in part by the fact that the low number of samples with 8% (11 of 131) mixed response, which suggests that a large number of mixed responses are necessary for the TL approach. Another possible reason might be the low number of metastases (5%, 12 of 235) from organs other than the lungs and lymph nodes. We believed that the lower 95% confidence interval of AUC (0.41 and 0.49 in the testing set) for NCE-radscore and CE-radscore indicated an insufficient predictive efficiency. NCE-radscore between the responders and non-responders had no significant difference in the testing set (*P*=0.23). Compared with the LL approach, the TL approach is inconvenient and time-consuming. Therefore, we have reasonably chosen the LL approach to further construct the combined-radiomics with NCE-radscore and CE-radscore.

Although radiomics based on single NCE-CT or CE-CT images are conventionally used for prediction, NCE-radiomics and CE-radiomics may contain complementary information regarding treatment response (31). To further improve the prediction effect of the radiomics model based on the baseline images, a combined-radiomics nomogram based on both NCE-radscore and CE-radscore for LL was built to predict response to immunotherapy. Excitingly, the combined-radiomics model showed better performance in predicting response to immunotherapy than the radiomics model based on single NCE-CT or CE-CT images, suggesting a potential complementarity between RFs based on NCE-CT and CE-CT images (31). In addition, the tumor boundaries of patients with advanced NSCLC are mostly unclear, so simple NCE-CT images cannot accurately delineate the tumor boundaries. Therefore, integrating radiomics features of NCE-CT and CE-CT images into a predictive panel as a radiomics model may be a robust approach for predicting response to immunotherapy. Compared our previous study (42), the combined-radiomics models based on only NCE/CE-CT images of baseline from the single largest lesion approach may be more promising for clinical application and early prediction.

What is unique about this study is that we conducted a single radiomics analysis based on NCE-CT and CE-CT images separately and combined-radiomics analysis based on NCE/CE-CT images. The results highlighted the feasibility and validity of the utility of combined-radiomics with NCE/CE-CT images analysis from the largest lesions. Meanwhile, we also conducted radiomics analysis on both NCE and CE for target lesions. Despite the poor results, it provides a meaningful indication for radiomics study of target lesions studies in immunotherapy. The next step is to construct a large sample composite model of target lesions from different organs, which may provide a consistent framework for RECIST1.1 and overcome the adverse effects of the mixed response pattern of immunotherapy in patients with NSCLC on radiomics features extraction.

Our study also possessed some limitations. First, the heterogeneity of the cohorts from multicenter, particularly for enhancement imaging parameters, could affect radiomics features extraction and analysis process, even though some efforts had been made to weaken the multicenter effect. Second, given that the fact of a limited number of complex responses and metastases outside the lung, the results of radiomics analysis for target lesions may be affected to some extent. Third, the combined-radiomics nomogram was not combined with currently known clinical biomarkers. Integrating data from different disciplines might construct a fully integrated model that can be applied to the clinical workflow. Fourth, the performance of clinical predictive models would decline over the continuous improvement of immunotherapy methods.

In conclusion, the results from our pilot study showed that the combined-radiomics nomogram incorporating the NCE/CE-CT images with the clinical factor of distant metastasis could serve as a non-invasive and cost-effective decision-support tool for better stratification of patients receiving immunotherapy with ICIs.

## Data Availability Statement

The original contributions presented in the study are included in the article/**Supplementary Material**. Further inquiries can be directed to the corresponding authors.

## Ethics Statement

The studies involving human participants were reviewed and approved by Tianjin Medical University Cancer Institute and Hospital. Written informed consent for participation was not required for this study in accordance with the national legislation and the institutional requirements.

## Author Contributions

Conception and design: MW, YaZ, YiL, and ZY. Literature research: MW, YaZ, and YiL. Collection and assembly of data: all authors. Clinical studies: MW, YaZ, and YiL. Data analysis and interpretation: MW, SW, and YG. Manuscript writing: MW and YaZ. All authors contributed to the article and approved the submitted version.

## Funding

This work was supported by the Chinese National Key Research and Development Project (2018YFC1315600), National Natural Science Foundation of China (81974277), and Demonstrative Research Platform of Clinical Evaluation Technology for New Anticancer Drugs (No. 2018ZX09201015).

## Conflict of Interest

Authors SW and YG were employed by company GE Healthcare China.

The remaining authors declare that the research was conducted in the absence of any commercial or financial relationships that could be construed as a potential conflict of interest.

## Publisher’s Note

All claims expressed in this article are solely those of the authors and do not necessarily represent those of their affiliated organizations, or those of the publisher, the editors and the reviewers. Any product that may be evaluated in this article, or claim that may be made by its manufacturer, is not guaranteed or endorsed by the publisher.
