# Supplementary material for: A Combined-Radiomics Approach of CT Images to Predict Response to Anti-PD-1 Immunotherapy in NSCLC: A Retrospective Multicenter Study
Source: Front Oncol. 2022 Jan 10;11:688679. doi: 10.3389/fonc.2021.688679 (PMC8784873; doi:10.3389/fonc.2021.688679)
Supplement: Supplementary file 1 [file DataSheet_1.docx]

**Supplementary material**

**Appendix S1: Inclusion and exclusion criteria**

Inclusion criteria:

1. histologically confirmed NSCLC; (2) immunotherapy with PD-1 ICIs at first or later line; (3) available pre-treatment demographics.

Exclusion criteria:

(1) CT images were of poor quality; (2) tumors were not clear after surgery; (3) time between baseline imaging and immunotherapy treatment exceeded four weeks; (4) unavailable NCE-CT or CE-CT images; (5) follow-up time from initiation of immunotherapy was less 6 months with regular clinical evaluations and CT scans after each 2 or 3 cycles of ICIs.

**Appendix S2: Inclusion criteria**

1. histologically confirmed stage NSCLC treated with platinum-based chemotherapy; (2) available pre-treatment demographics; (3) both NCE-CT and CE-CT examinations were done within a four-week period before treatment; (4) clear tumor boundaries; (5) follow-up time from initiation of chemotherapy was greater than 6 months.

**Appendix S3: CT scanning protocol**

Scanning parameters were as follows:

1. 120 kVp with tube current adjusted automatically

2. Reconstruction thickness:

1.25 mm for Discovery CT750 HD and Discovery CT750 scanner

3 mm for Siemens SOMATOM Definition AS+ scanner

4 mm for Canon Aquilion ONE scanner

5 mm for the other five scanners

3. Field of view (FOV): 36.5 cm to 44 cm

4. Matrix: 512×512

5. Reconstruction Kernel: Standard Algorithm (Std for GE, B30f for Siemens, or other equivalent)

**Appendix S5: Equations**

Equation 1:

Radscore = -0.159 × original_glszm_GrayLevelVariance -

0.186 × wavelet_LLH_glcm_ClusterShade - 0.085 × original_shape_Elongation + 0.036 × log_sigma_3_0_mm_3D_glszm_LargeAreaLowGrayLevelEmphasis -

0.094 × wavelet_LHL_glcm_MCC + 0.049 × wavelet_LHH_glcm_ClusterShade + 0.285 × wavelet_LHL_glrlm_LongRunHighGrayLevelEmphasis +

0.215 × wavelet_HLH_firstorder_Median - 0.206

Equation 2:

Radscore = -0.056 × wavelet_HLL_glcm_ClusterTendency -

0.079 × wavelet_HLH_glcm_Correlation + 0.092 × lbp_3D_k_ngtdm_Busyness + 0.062 × wavelet_LLL_glcm_MaximumProbability - 0.335

Equation 3:

Radscore = -0.107 × wavelet_LHH_firstorder_Skewness +

0.053 × wavelet_LHH_glszm_SizeZoneNonUniformityNormalized - 0.2

Equation 4:

Radscore = -0.222 × wavelet_HLL_gldm_DependenceEntropy +

0.185 × wavelet_HLH_firstorder_Skewness -

0.164 × wavelet_HHH_glszm_SmallAreaLowGrayLevelEmphasis +

0.109 × wavelet_HHL_glcm_ClusterShade -

0.041 × wavelet_HHH_glszm_SizeZoneNonUniformityNormalized +

0.08 × lbp_3D_k_firstorder_10Percentile +

0.121 × wavelet_HLL_glszm_LargeAreaLowGrayLevelEmphasis - 0.297

Equation 5：

Radscore = 0.435 × Equation 1 + 1.154 ×Equation 2 - 0.648

**Table S1.** 1316 radiomics features extracted from baseline NCE-CT and CE-CT images using Artificial Intelligence Kit software version 3.3.0 (GE Healthcare, China)

| **Feature Category** | **Number of features** | **Feature Name** |
| --- | --- | --- |
| First Order Histogram Features | 18 | 1.Energy |
|  |  | 2.Total Energy |
|  |  | 3.Entropy |
|  |  | 4.Minimum Intensity |
|  |  | 5.The 10th percentile of X |
|  |  | 6.The 90th percentile of X |
|  |  | 7.Maximum Intensity |
|  |  | 8.Mean Intensity |
|  |  | 9.Median Intensity |
|  |  | 10.Range |
|  |  | 11.Mean Absolute Deviation (MAD) |
|  |  | 12.Robust Mean Absolute Deviation (rMAD) |
|  |  | 13.Root Mean Squared (RMS) |
|  |  | 14.Skewness |
|  |  | 15.Kurtosis |
|  |  | 16.Variance |
|  |  | 17.Uniformity |
|  |  | 18.Interquartile range |
| Shape-related Statistics | 14 | 1.Volume |
|  |  | 2.Surface Area |
|  |  | 3.Surface Volume Ratio |
|  |  | 4.Sphericity |
|  |  | 5.Voxel Number |
|  |  | 6.Maximum 3D diameter |
|  |  | 7.Maximum 2D diameter (Slice) |
|  |  | 8.Maximum 2D diameter (Column) |
|  |  | 9.Maximum 2D diameter (Row) |
|  |  | 10.Major Axis Length |
|  |  | 11.Minor Axis Length |
|  |  | 12.Least Axis Length |
|  |  | 13.Elongation |
|  |  | 14.Flatness |
| Gray Level Co-occurrence Matrix (GLCM) | 24 | 1.Autocorrelation |
|  |  | 2.Joint average |
|  |  | 3.Cluster Prominence |
|  |  | 4.Cluster Tendency |
|  |  | 5.Cluster Shade |
|  |  | 6.Contrast |
|  |  | 7.Correlation |
|  |  | 8.Difference Average |
|  |  | 9.Difference Entropy |
|  |  | 10.Difference Variance |
|  |  | 11.Joint energy |
|  |  | 12.Joint entropy |
|  |  | 13.Informational measure of correlation 1 |
|  |  | 14.Informational measure of correlation 2 |
|  |  | 15.IDM (inverse difference moment) |
|  |  | 16.IDMN (inverse difference moment normalized) |
|  |  | 17.ID (inverse difference |
|  |  | 18.Inverse difference normalized |
|  |  | 19.Inverse variance |
|  |  | 20.MCC |
|  |  | 21.Maximum Probability |
|  |  | 22.Sum Average |
|  |  | 23.Sum Entropy |
|  |  | 24.Sum of Squares |
| Gray Level Size Zone Matrix (GLSZM) | 16 | 1.SAE (small area emphasis) |
|  |  | 2.LAE (large area emphasis) |
|  |  | 3.GLN (gray level non-uniformity) |
|  |  | 4.GLNN (gray level non-uniformity normalized) |
|  |  | 5.SZN (size zone non-uniformity) |
|  |  | 6.SZNN (size zone non-uniformity normalized) |
|  |  | 7.ZP (Zone Percentage) |
|  |  | 8.Gray level variance (GLV) |
|  |  | 9.Zone Variance (ZV) |
|  |  | 10.Zone Entropy (ZE) |
|  |  | 11.LGLZE (low gray level zone emphasis) |
|  |  | 12.HGLZE (high gray level zone emphasis) |
|  |  | 13.SALGLE (small area low gray level emphasis) |
|  |  | 14.SAHGLE (small area high gray level emphasis) |
|  |  | 15.LALGLE (low area low gray level emphasis) |
|  |  | 16.LAHGLE (low area high gray level emphasis) |
| Gray Level Run Length Matrix (GLRLM) | 16 | 1.SRE (Short Run Emphasis) |
|  |  | 2.LRE (Long Run Emphasis) |
|  |  | 3.GLN (Gray Level Non-uniformity) |
|  |  | 4.GLNN (Gray Level Non-uniformity Normalized) |
|  |  | 5.RLN (Run Length Non-uniformity) |
|  |  | 6.RLNN (Run Length Non-uniformity) |
|  |  | 7.RP (Run Percentage) |
|  |  | 8.GLV (Gray Level Variance) |
|  |  | 9.RV (Run Variance) |
|  |  | 10.RE (Run Entropy) |
|  |  | 11.LGLRE (low gray level run emphasis) |
|  |  | 12.HGLRE (high gray level run emphasis) |
|  |  | 13.SRLGLE (short run low gray level emphasis) |
|  |  | 14.SRHGLE (short run high gray level emphasis) |
|  |  | 15.LRLGLRE (long run low gray level emphasis) |
|  |  | 16.LRHGLRE (long run high gray level run emphasis) |
| Neigbouring Gray Tone Difference Matrix (NGTDM) | 5 | 1.Coarseness |
|  |  | 2.Contrast |
|  |  | 3.Busyness |
|  |  | 4.Complexity |
|  |  | 5.Strength |
| Gray Level Dependence Matrix (GLDM) | 14 | 1.SDE (Small Dependence Emphasis) |
|  |  | 2.LDE (Large Dependence Emphasis) |
|  |  | 3.GLN (Gray Level Non-Uniformity) |
|  |  | 4.DN (Dependence Non-Uniformity) |
|  |  | 5.DNN (Dependence Non-Uniformity Normalized) |
|  |  | 6.GLV (Gray Level Variance) |
|  |  | 7.DV (Dependence Variance) |
|  |  | 8.DE (Dependence Entropy) |
|  |  | 9.LGLE (Low Gray Level Emphasis) |
|  |  | 10.HGLE (High Gray Level Emphasis) |
|  |  | 11.SDLGLE (Small Dependence Low Gray Level Emphasis) |
|  |  | 12.SDHGLE (Small Dependence High Gray Level Emphasis) |
|  |  | 13.LDLGLE (Large Dependence Low Gray Level Emphasis) |
|  |  | 14.LDHGLE (Large Dependence High Gray Level Emphasis) |
| Wavelet transform | 744 | Wavelet features |
| LoG transform | 186 | Laplacian (LoGsigma=2.0/3.0) features |
| LBP transform | 279 | Local Binary Pattern features |

**Table S2.** Demographics and clinical characteristics of the chemotherapy cohorts, categorized by responders and nonresponders

| **Variables** | **Sample** | **Responders** | **Non-responders** | ***P Value*** |
| --- | --- | --- | --- | --- |
| Age, madian |  | 60 (56, 66) | 58 (51, 63) | 0.17 |
| Sex, No. (%) |  |  |  | 0.30 |
| Male | 26 | 18 (75.00%) | 8 (100.00%) |  |
| Female | 6 | 6 (25.00%) | 0 (0.00%) |  |
| Smoking history, No. (%) |  |  |  | 1.0 |
| Non-smokers | 6 | 5 (20.83%) | 1 (12.50%) |  |
| Smokers | 26 | 19 (79.17%) | 7 (87.50%) |  |
| Pathological type, No. (%) |  |  |  | 1.0 |
| Adenocarcinoma | 24 | 18(75.00%) | 6(75.00%) |  |
| Others | 8 | 6(25.00%) | 2(25.00%) |  |
| Distant metastasis, No. (%) |  |  |  | 0.15 |
| Absence | 7 | 7(29.17%) | 0(0.00%) |  |
| Presence | 25 | 17(70.83%) | 8(100.00%) |  |


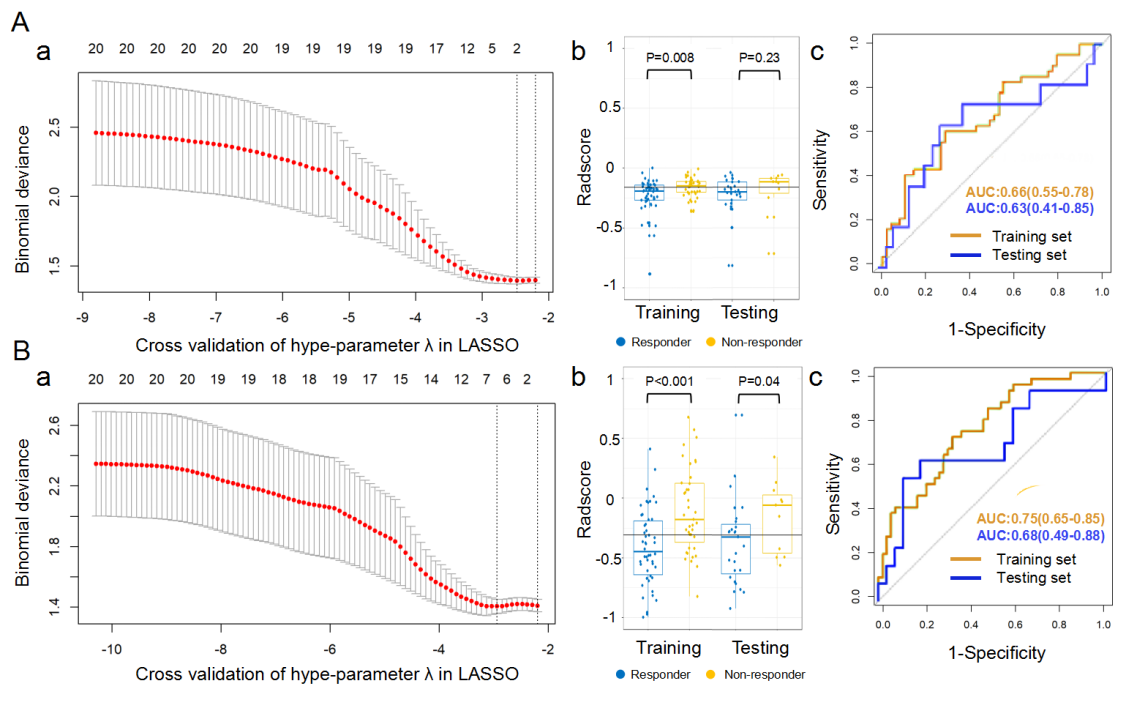


**Figure S1.** Performance of the NCE-radiomic signature (A) and CE-radiomic signature (B) from target lesions approach in training and testing sets. (Aa, Ba) Tuning penalty factor (λ) in the LASSO model used 10-fold cross-validation via minimum criteria. The binomial deviance metrics (the y-axis) were plotted against log(λ) (the upper x-axis) and the number of selected features (the bottom x-axis). (Ab, Bb) Box and whisker plots depict radscore comparison between responders and non-responders. (Ac, Bc) ROC curves of radiomics signatures in training and testing sets.

**Table S3.** ROC analysis for the NCE-radiomics, CE-radiomics and combined-radiomics models from largest lesion approach in the chemotherapy cohorts

| **Variables** | **NCE-CT** | **CE-CT** | **Combining** |
| --- | --- | --- | --- |
| Radscore |  |  |  |
| AUC (95% CI) | 0.61  (0.36-0.87) | 0.49  (0.22-0.77) | 0.57  (0.31-0.84) |
| *P* | 0.98 | 0.69 | 0.17 |
| Specificity | 0.87 | 0.25 | 0.63 |
| Sensitivity | 0.25 | 0.63 | 0.50 |
| Accuracy (95% CI) | 0.72  (0.53-0.86) | 0.34  (0.19-0.53) | 0.59  (0.41-0.76) |
| Nomogram |  |  |  |
| AUC (95% CI) | 0.69  (0.46-0.92) | 0.64  (0.41-0.86) | 0.61  (0.36-0.87) |
| Specificity | 0.92 | 0.83 | 0.91 |
| Sensitivity | 0.37 | 0.50 | 0.33 |
| Accuracy (95% CI) | 0.59  (0.41-0.76) | 0.75  (0.56-0.88) | 0.53  (0.35-0.71) |
